# Metagenomics Study Reveals Changes in Gut Microbiota in Centenarians: A Cohort Study of Hainan Centenarians

**DOI:** 10.3389/fmicb.2020.01474

**Published:** 2020-07-02

**Authors:** Zhe Luan, Gang Sun, Yun Huang, Yunsheng Yang, Ruifu Yang, Congyong Li, Tingting Wang, Di Tan, Shirui Qi, Chen Jun, Cong Wang, Shufang Wang, Yiming Zhao, Yujia Jing

**Affiliations:** ^1^Chinese PLA General Hospital, Beijing, China; ^2^Realbio Technology Co., Ltd., Hangzhou, China; ^3^Academy of Military Medical Sciences, Beijing, China; ^4^Tianjin First Central Hospital, Tianjin, China; ^5^Hainan Branch of Chinese PLA General Hospital, Sanya, China

**Keywords:** centenarians, gut microbiota, longevity, metagenomics, aging

## Abstract

Several studies based on 16SrDNA analysis have revealed certain unique characteristics of gut microbiome in centenarians. We established a prospective cohort of fecal microbiota and conducted the first metagenomics-based study among centenarians. The objective was to explore the dynamic changes of gut microbiota in healthy centenarians and centenarians approaching end of life and to unravel the characteristics of aging-associated microbiome. Seventy-five healthy centenarians residing in three regions of Hainan participated in follow-up surveys and collection of fecal samples at intervals of 3 months. Data pertaining to dietary status, health status scores, cause of disease and death, and fecal specimens were collected for 15 months. Twenty participants died within 20 months during the follow-up period. The median survival time was 8–9 months (range, 1–17) and the mortality rate was 14.7% per year. The health status scores before death were significantly lower than those at 3 months before the end of the follow-up period [median score: 3 (range, 1–5), *P* < 0.05]. At this time, the participants mainly exhibited symptoms of anorexia and reduced dietary intake and physical activity. Metagenomics sequencing and analysis were carried out to characterize the gut microbiota changes in the centenarians during their transition from healthy status to death. Anosim analysis showed a significant change in gut microbiota from 7 months prior to death (*R* = 0.10, *P* = 0.02). All participants were grouped with 7 months before death as cut-off; no significant difference in α diversity was found between the two groups (*P* = 0.45). Semi-supervised monitoring and log rank sum analysis revealed significant changes in the abundance of ten bacterial species before death; of these, eight species were significantly reduced (*Akkermansia muciniphila*, *Alistipes finegoldii*, *Alistipes shahii*, *Bacteroides faecis*, *Bacteroides intestinalis*, *Butyrivibrio crossotus*, *Bacteroides stercoris*, and *Prevotella stercorea*) while two were significantly increased before death (*Bifidobacterium longum* and *Ruminococcus bromii*). Compared to centenarians in northern Italy, Hainan centenarians exhibited unique characteristics of gut microbiome. The abundance of ten bacterial species showed significant changes starting from 7 months before death. We speculate that these changes might occur before the clinical symptoms of deterioration in health status.

## Introduction

According to a survey published by the United Nations in 2013, the proportion of people over the age of 80 years account for 14% of the global population, and this proportion is predicted to exceed 19% by the year 2050; at that time, the number of elderly population are projected to increase to 390 million, about three times the number of current population (Cohen, 2003). The global phenomenon of population aging is liable to lead to an increased incidence of age-related diseases. Thus, improvement in the health status of elderly individuals and maintenance of the quality of life in the latter years of life is a key research imperative. Experimental studies investigating the mechanism of longevity have extensively employed *Caenorhabditis elegans*, zebrafish, and Drosophila as animal models owing to their favorable attributes, such as short lifespan, simple model construction, and convenient intervention. Several studies have indicated that gut microbiota plays a major role in contributing to longevity of the elderly (Kenyon, 2010; Sonowal et al., 2017; Catterson et al., 2018). With the highest level of human lifespan, centenarians represent the best model of “successful” aging owing to the relatively lower incidence of chronic diseases, lower mortality, and prolonged healthy lifespan. Identification of the unique dominant gut microbiota in centenarians is likely to facilitate a better understanding of the mechanism of longevity. However, due to the low population of centenarians, their wide spatial distribution, the difficulty in collecting and preserving fecal specimens, and the high costs of metagenomics sequencing, previous studies on the gut microbiota of centenarians have largely been cross-sectional studies. Till date, cohort studies of the dynamic changes in microbiome have been relatively rare and none of these studies have employed metagenomics analysis (Kenyon, 2010; Santoro et al., 2018). The symbiotic relationship between the human body and the gut microbiota is widely recognized (Lopez-Otin et al., 2016). The changes in gut microbiota are closely related to human health status. Several factors affect the changes in gut microbiota, including individual differences, population-level differences, and environmental variables (Rothschild et al., 2018). Interactions within the symbiotic ecosystem throughout the life span lead to significant diversity and inter-individual differences. This adaptability of gut microbiota helps modulate the host immune and metabolic pathways in response to individual requirements, and has a profound impact on the health status and biological processes (Santoro et al., 2018). The gut microbiota of centenarians has its unique advantages. Studies have shown a positive effect of probiotics on maintenance of health including an anti-aging effect (Heintz and Mair, 2014; Kim et al., 2016). Metabolomic analysis of blood and urine samples of 143 elderly people (30 males and 113 females; average age: 100.9 ± 2.1 years) in northern Italy showed significant differences between the elderly and the young elderly groups with respect to the metabolism of lipids, amino acids, and gut microbiota (Collino et al., 2013). The altered lipid metabolism in the elderly group suggests the unique ability of the healthy elderly individuals to respond to oxidative stress and chronic inflammation. The results of detailed comparative studies indicated that this anti-oxidative ability is positively correlated with some *Proteobacterial* genera (Biagi et al., 2010).

Aging is associated with altered profile of gut microbiota. The dominant gut microbiota in elderly may be associated with longevity. The gut microbiota of elderly are more adaptable to accept opportunistic and symbiotic bacteria. Studies have shown that high abundance and enrichment of health-associated bacteria such as *Akkermansia*, *Bifidobacterium*, and *Christensenellaceae* may be related to longevity (Biagi et al., 2016). However, the gut microbiota perpetually evolves over time, from young age to old age with declining health status. Identification of gut microbiome at a certain time-point cannot fully unravel the secrets of longevity. Moreover, the gut microbiota in dying centenarians are not well characterized (Faith et al., 2013; Franzosa et al., 2014; Abu-Ali et al., 2018). Thus it is of great value to identify the gut microbiota that play a pivotal role in longevity, especially in the context of deteriorating health status of centenarians.

Based on the results of previous studies, we speculate that the changes in gut microbiota in centenarians might come before the changes in the overt health status. Metagenomics sequencing allows for accurate classification of microbiota up to species level as well as the annotation and analysis of the bacteria at the functional level without direct functional prediction (Gosalbes et al., 2012; Lynch and Pedersen, 2016). In this study, we selected the Hainan province to set up a dynamic follow-up system. Hainan is an island with a relatively closed location which is less disturbed by external environment; in addition, the local populace exhibits considerable homeogeneity with respect to the sociocultural milieu and the dietary habits. Hainan province was recognized by the International Expert Committee of Population Aging and Longevity as a World Longevity Island on August 27, 2014 for its highest percentage of centenarians (18.75/100,000) in China (Wang et al., 2016). Hainan exhibits the attributes that are conducive for microbial ecology study. These include a relatively large sample of subjects with extreme phenotype (population distribution), genetic homogeneity, and lifestyle consistency. These characteristics make it particularly suitable for identification of gut microbiota associated with longevity. A large-scale cohort study of centenarians in Hainan has been performed by the Hainan Branch of the General Hospital of the People’s Liberation Army (PLAGH) (He et al., 2018). The study covered about 80% of the centenarians residing in the island (*n* = 1,473). Based on the previous data, we randomly selected 75 healthy centenarians from Chengmai, Danzhou, and Lingao districts for this nested cohort study. Fecal samples were collected every 3 months until the death of the participant. Subsequently, metagenomics sequencing and analysis were carried out to characterize the gut microbiota changes in the centenarians during their transition from healthy status to death. In particular, we sought to determine the predominantly changed bacteria which may have strong influence on the decline in health and the eventual death.

## Materials and Methods

### Health Status, Scores of Body Status, and Mortality Rate of Centenarians in Hainan Province

This study was approved by the Ethics Committee (Ethics No. 301hn11-2017-03). All centenarians enrolled in the study voluntarily agreed to participate in the study. Written informed consent was obtained from all subjects prior to their enrollment. The term “Centenarians” is used to refer to elderly individuals who were born prior to January 1, 1917 and who had resided in Hainan for more than 20 years (date of birth was collected from ID card or registered permanent residence). The cohort analysis is based on the nested study of China Hainan Centenarian Cohort Study (CHCCS) (He et al., 2018). The study design refers to some previous standardized cohort studies conducted in China, such as Guangzhou Biobank, China Kadoorie Biobank, and China Health and Retirement Longitudinal Study (CLHLS). A total of 146 centenarians from Hainan province participated in the baseline multidisciplinary assessment of health status.

The first round of centenarians’ survey included questionnaire for baseline survey, collection of biological specimens, and physical and laboratory examination. The questionnaire survey was conducted by nurses who were conversant with the Hainan dialect.

Physical health assessment was performed by a multidisciplinary team of specialists and nurses who had more than 5 years’ experience in their respective specialties (cardiology, ultrasound, otorhinolaryngology, gynecology, and obstetrics). The components of assessment included medical history, general physical examination, electrocardiogram, collection of blood samples, ultrasound examination of heart, abdomen, vagina, and joints. Excluding clear diseases, the results of physical examination are healthy (Tables 1, 2). Seventy-five healthy centenarians without any of the clear diseases were selected for this study. The average age of subjects was 103.1 ± 2.8 years (range, 96–113); 59 (78.7%) participants were women.

**TABLE 1:** Multidisciplinary health examination for comprehensive assessment of the physical status of centenarians.

**Specialty**	**Examination items**
Cardiology	ECG, echocardiography, macrovascular ultrasound
General surgery	General physical examination
Dental	Oral examination and brushing examination of oral mucosal cells
Otolaryngology	Otolaryngology examination, hearing, and acoustic impedance examination (MADESEN Xeta)
Gastroenterology	Digital rectal examination
Ultrasonography	Ultrasound of liver, gallbladder, pancreas, spleen, kidneys, and knee joint
Gynecology	Gynecological examination and vaginal exfoliative cytology
Laboratory	Blood routine, blood biochemical, blood coagulation, tumor markers, infectious disease serology, and immunology related indices
	

**TABLE 2:** The following diseases were excluded for the healthy centenarians in this study.

**Domains**	**Comprehensive health measures**
Endocrine	1. Diabetes
	2. HbA1C > 6.5%
	3. Thyroid diseases
Cardiovascular	4. Hypertension
	5. Systolic BP > 140 mmHg
	6. Diastolic BP > 90 mm/Hg
	7. Rapid pulse > 80 bpm
	8. Heart attack
	9. Cerebrovascular disease
	10. Heart failure
Lung	11. COPD
	12. Asthma
Immune	13. Arthritis
	14. Peptic ulcer
Filtration	15. Chronic kidney disease
	16. Severe liver damage
Cancer	17. Skin
	18. Reproductive
	19. Non-reproductive

Follow-up visits were conducted regularly at 3-monthly intervals by professional clinicians to investigate the changes in the physical status (including the final cause of death), appetite and food intake of the centenarians through in-person interviews. Stool samples were collected until the death of the subjects. The inclusion criteria were: (1) absence of impaired consciousness, serious organic diseases, or major digestive tract diseases; (2) no history of trauma or surgery in the last 2 years; (3) no history of treatment with antibiotics, acid inhibitors, laxatives, probiotics, prebiotics, or other drugs that may affect gut microbiota in the 3-month period immediately preceding the sample collection (Cammarota et al., 2017); (4) no other anal disease.

The exclusion criteria were: (1) results of health examination do not qualify the health criteria; (2) Refusal of patients or their families to cooperate; (3) patients with loss of contact in five cycles of follow-up.

Health status scores: Due to the unique health status of centenarians, the health survey form was formulated taking cognizance of the potential inconvenience caused by medical treatment and clinical examination. According to their overall health status, the participants were grouped into five grades: totally healthy; general healthy; infirmity; sickbed; self-care inability; and articulo mortis (the corresponding subjective scores were 1–5, respectively).

### Fecal Sample Collection and Preservation

Fresh feces were collected from each subject on the day of examination by independent defecation or via digital rectal examination. Each sample of fresh feces (5 g) was labeled and preserved in 2 mL of RNAlater Stabilization Solution (Sigma) and transported using dry ice. On arrival at the laboratory all fecal samples were stored at −80°C until further use (Anderson et al., 2016; Claesson et al., 2017; Vogtmann et al., 2017).

Fecal samples were labeled with “3-digit number + volunteer name + sampling date.” The three-digit number was composed of “number given on first follow-up – round number – source of samples.” The source of fecal samples from centenarians was labeled as C, and the centenarians’ gender was labeled as F or M for female and male centenarians, respectively. All fecal samples were sent for metagenomic sequencing and analysis.

### Metagenomic Sequencing

DNA was extracted from each fecal sample using improved protocol based on the manual of QIAamp Fast DNA Stool Mini Kit (Qiagen, Germany). In detail, 1 mL of InhibitEX Buffer and adequate amount of glass beads (0.5 mm diameter, Qiagen) were added to each 200 mg of feces. The mixture was homogenized twice with a Homogeneous instrument (FASTPREP-24, Aosheng Biotech, China) for I min each at a frequency of 60 Hz. Subsequently, DNA purification was performed according to the manufacturer’s instructions. DNA paired-end libraries with an insert size of 500 bp were prepared following the Illumina TruSeq DNA Sample Prep v2 Guide (Illumina, Inc., San Diego, CA, United States). Quality of all libraries was evaluated using an Agilent bioanalyzer (Agilent Technologies, Wokingham, United Kingdom) and the DNA LabChip 1,000 kit. All samples were subject to 150 bp paired-end sequencing using an Illumina HiSeq 2500 sequencer (Illumina, Inc., San Diego, CA, United States). Raw reads were filtered to trim nucleotides from the 3′ end using a quality threshold of 30; adaptors and low-quality reads (i.e., reads containing more than 50% nucleotides below Q30, reads short than 70 bp) were removed, as described elsewhere (Zi et al., 2018). Host reads were removed by alignment to hg38 using SOAP2.21 (Li et al., 2008). An average of 96.28% high quality reads were obtained from all samples and used in the subsequent analysis.

### *De novo* Assembly and Gene Catalog Construction

SOAP *de novo* (version 2.04) based on the De-Brujin graph algorithm was used to assemble the high quality reads into contigs. Open reading frames (ORFs) were predicted later using MetaGeneMark (version 3.38) (Hideki et al., 2006). Pairwise comparisons of predicted ORFs (filtered with a length of 100 bp) were performed to obtain a non-redundant gene set using CD-HIT (version 4.5.7) at 95% identity and 90% coverage (Li, 2006). The final non-redundant gene catalog contained 6,655,439 microbial genes with an average length of 682 bp. Functional annotations were carried out by BLASTP search against the Kyoto Encyclopedia of Genes and Genomes (KEGG) database (Minoru et al., 2004).

### Taxonomic and Gene Profiling

Microbial composition at each taxonomic level and gene abundance were determined by MetaPhlAn2 (version 2.5.0) with default parameters. All high quality reads were aligned against the non-redundant gene catalog built above using SOAPalign 2.21 with parameters of “−r 2 −m 100 − × 1,000.” Only reads with both paired ends mapped to a gene were used in the analysis (Qin et al., 2014).

### MGS Analysis

Genes were clustered into metagenomic species (MGS) according to the method described by Le Chatelier et al. (2013) and Nielsen et al. (2014). First, the differentially abundant genes (*P* < 0.05, Wilcoxon test) with a pairwise Spearman correlation coefficient (rho) >0.8 were clustered using single-linkage clustering according to their abundance variation across all samples. Secondly, the mean abundance of each cluster containing more than 25 genes was computed, and clusters with pairwise rho >0.8 were fused as MGS. The taxonomic annotation and abundance profile of each MGS were generated according to the taxonomy and the relative abundance of the corresponding genes. Taxon of each MGS was assigned as below: (1) >90% of the genes in this MGS were assigned to the same taxon; (2) the best hit to the same phylogenetic group (>95% identity and >90% overlap of query).

### Quantitative Real-Time PCR

Targeted species between different fecal samples were detected by Quantitative Real-Time PCR Thermal Cyclers (Germany), operated according to the handbook of TUREscript 1st Stand cDNA SYNTHESIS Kit to synthesize cDNA. Primers were designed by Beacon Designer 7.9. After adding all components, samples were centrifuged at 6,000 rpm for 1 min to keep all components in the bottom [reaction condition Step 1 – 95°C for 3 min, Step 2 – 95°C for 10 s, Step 3 – 58°C for 30 s + plate read, Step 5 – go to Step 2, 39 cycles, and Step 6 – melt curve analysis (60–95°C + 1°C/cycle, holding time 4 s)]. The relative gene expression was calculated by the qPCR soft3.2 software automatically. The software was designed based on Pfaffl method (see Supplementary Material for further details).

### Time Sequence

The increase or decrease of species in different time periods was expressed as positive or negative area under the curve. If the species shows an increase in a certain time period, the area under the curve is positive; similarly, if the species shows a decline in a certain time period, the area under the curve is negative. Using the following four rules, we identified the bacterial species with inflection point before death, and which showed the same trend in 80% of individuals. These bacterial species may have similar functions and have a critical inflection time point before death. (1) We calculated the positive and negative area of each individual at two consecutive time-points; all positive and all negative bacteria are accepted as candidate bacteria. If there were different bacteria, the second condition was assessed; (2) the average area for each individual was calculated at two consecutive time-points. The bacteria meet the positive/negative (take large value) area mean is greater than the negative/positive area mean. Bacteria associated with value >1.5 times were accepted as candidate bacteria; if less than 1.5 times, the third condition was assessed; (3) We calculated the maximum triangular monthly span of each individual at two consecutive time points; if <5%, these bacteria were accepted as candidate bacteria; if not, the fourth condition was assessed; (4) We calculated the first one of each individual bacteria. If the difference between the abundance at the time-point and that at the preceding sampling time-point occupied more than 30% of the total figure, the bacteria were accepted as candidate bacteria; if it was smaller, the bacteria were filtered out.

## Results

### Description of Metagenomic Analysis

Data pertaining to 191 fecal samples from 75 Hainan healthy centenarians were included in the dynamic cohort analysis (centenarians died at different times, therefore not all of them could successfully collect five samples; only 191 stool samples were collected in total). All participants were determined to be healthy centenarians by multidisciplinary examination and had resided in the Hainan Province for more than 20 years (average age: 103.1 ± 2.8 years). Fifty-nine (78.7%) centenarians were female. The follow-up survey lasted for 20 months; 20 participants died during this period, the median survival time was approximately 8–9 months, and the one-year mortality rate was 14.7%. By comparing the health status scores, we found that the median time of significant decline in the physical condition of the subjects was about 3 months. All subjects died at their home in this study due to the scattered residence. All centenarians who participated in the survey received financial subsidy from the local civil affairs, which was paid by a specially designated person on a monthly basis. Moreover, it was checked whether the old people were still alive; therefore, the timing of deaths were available for all subjects. However, no official death certificate was available for all subjects. The follow-up survey indicated that the main symptoms of the centenarians before death were anorexia and reduced dietary intake. None of the participants died of unnatural causes.

Microarray-based genomic selections for gut microbiome sequencing were carried out using Hiseq2500; about 13.44 ± 3.82 Gb data was obtained from each sample on average. Moreover, phenotypic information of these centenarians were collected on the first round, including dietary and living habits, past medication history, and information from multidisciplinary health examination. Through quality control and removal of sequence reads mapping to the human genome by using MetaPhlAn 2.0, the results of microbiome sequencing were mapped to about 1 million genes of microbial taxonomic specific marker to predict the relative abundance of different microbiota. For each participant, we predicted abundance of 1,361 microbial taxonomic clades, ranging from four phyla to 534 species. Most of the reads were from bacteria (97.96%), 0.22% were from archaea, 1.81% were from viruses, and the last 7.55e-05% pertained to eukaryotes. *Bacteroidetes* and *Firmicutes* were the two dominant phyla at the phyla level, while *Escherichia* and *Bacteroides* were the two dominant genera at the genus level (*Escherichia coli* and *Prevotella copri* at the species level). The fecal metagenome of 191 longevity individuals was sequenced using Illumina platform. Finally, 6,655,439 microbial genes were obtained, with an average length of 682 bps.

### Microbiome Changes Cut-Off in 7 Months Before Death

We assumed that the changes in gut microbiota might come before the decline in the health status of centenarians. Thus, based on the metagenomic analysis of fecal samples, we used continuous dichotomy method to organize the fecal samples with different time-points. We identified the time inflection point of significant changes in gut microbiome by using bioinformatics analysis.

**FIGURE 1:**
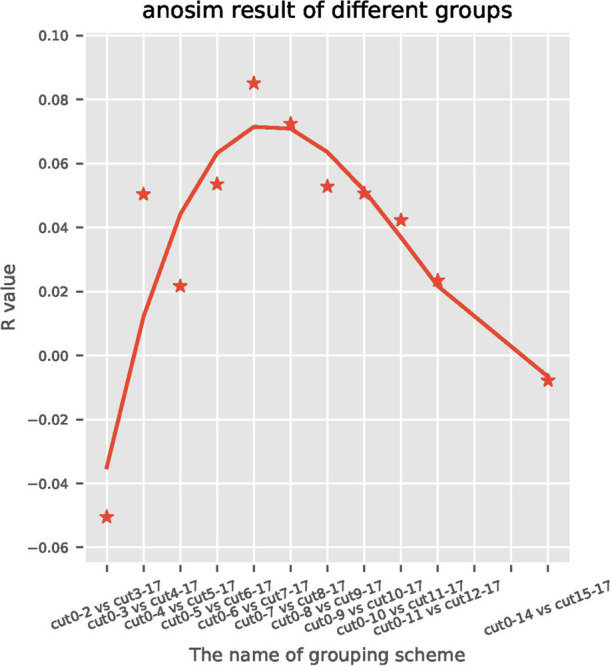
Anosim analysis is a statistical method used to analyze the significant differences between high-dimensional data of each group and to assess the integrity and significance of the differences between two groups. A positive *R* value indicates a significant difference between each group and the difference depends on the absolute value of *R*. The horizontal axis represents the month of death, the vertical axis represents the *R* value, and each spot represents the difference of *R* value between the two groups corresponding to different months of death. The most significant difference (*R* = 0.10, *P* = 0.02) was observed in the cut seven sub-group, which indicates that the 7th month was the inflection point of the gut microbiota changes.

A total of 191 sequentially collected fecal samples of centenarians were arranged with the order of time gap ranging from the time of death and the time of stool collection (range: 0–17 months). For the subjects who were still alive at the end of the follow-up, the time gap was calculated by using the last follow-up time to subtract the time of stool collection. Then the samples were divided into two groups using Nth month before death as the cut-off point. Taking Nth for 7th as an example, fecal samples collected earlier than 7 months were classified as cut 7–17 group, while the remaining samples collected within 6 months before the death were classified as cut 0–6 group; this generated a total of 11 groupings (Figure 1). Anosim analysis was performed on each group to find the most significant cut-off point of the difference between the gut microbiome of centenarians. We found that the most significant difference (*R* = 0.10, *P* = 0.02) between two groups occurred at the 7th month before death, which was defined as the possible time inflection point during dying period. Therefore, the time inflection point of the gut microbiota changes during the transition from healthy state to weak state could be predicted, i.e., the 7th month before death.

### Characteristics of Gut Microbiota in Healthy Centenarians and Dying Centenarians

**FIGURE 2:**
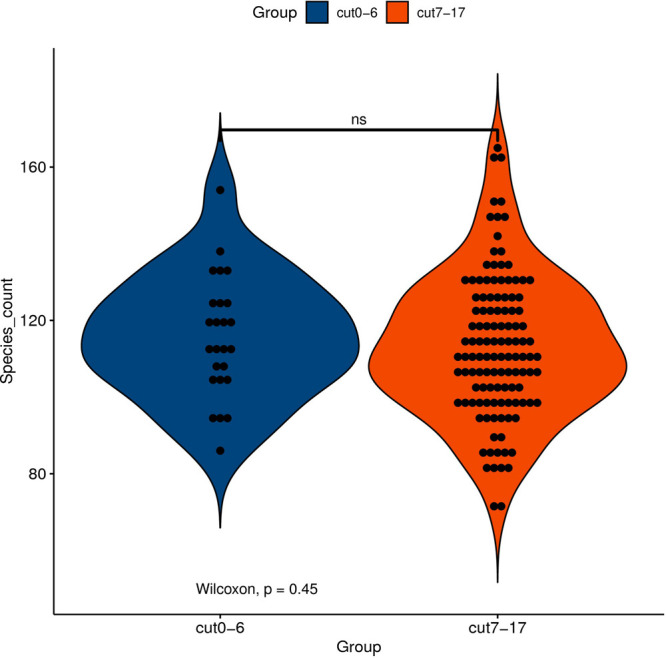
The abscissa represents the grouping of samples, and the ordinate represents the number index of species under different grouping. The external shape is the density distribution, and the internal black spot represents the position of the sample. Asterisk (*) indicates significant difference (0.01 < *P* < 0.05); double asterisk (**) indicates extremely significant difference (*P* < 0.01); NS indicates no significant difference.

Although the gut microbiota changes of dying centenarians can be grouped by α diversity analysis, there was no significant difference between each group (Figure 2). Semi-supervised learning and log rank sum analysis revealed significant changes in the abundance of 10 different species; eight of these showed a significant decrease in abundance (*Akkermansia muciniphila*, *Alistipes finegoldii*, *Alistipes shahii*, *Bacteroides faecis*, *Bacteroides intestinalis*, *Butyrivibrio crossotus*, *Bacteroides stercoris*, and *Prevoa stercorea*), while two species were significantly enriched before death (*Bifidobacterium longum* and *Ruminococcus bromii*). Several different methods were used to identify the differentially abundant bacteria (Table 3).

**TABLE 3:** The 10 differentially expressed species and the corresponding analysis method.

**S. No.**	**Changed species**	**Analysis method**
1	*Akkermansiamuciniphila*	Time sequence
2	*Alistipesfinegoldii*	Time sequence
3	*Alistipesshahii*	J-T non-parametric test
4	*Bacteroidesfaecis*	Time sequence
5	*Bacteroidesstercoris*	J-T non-parametric test
6	*Bacteroides intestinalis*	Time sequence
7	*Butyrivibriocrossotus*	Time sequence
8	*Prevotellastercorea*	Lefse analysis
9	*Bifidobacterium longum*	Lefse analysis, J-T non-parametric test
10	*Ruminococcusbromii*	Time sequence

### Dietary Survey

We investigated the dietary pattern of 27 out of the 75 centenarians who were found to have a healthy status in the first round of multidisciplinary health examination. Follow-up visits were conducted by professional clinicians to investigate the changes in the physical status (including the final cause of death), appetite, and food intake of the centenarians through in-person interviews. The results revealed no significant changes in the physical status, food intake, or appetite of the centenarians before and after 7 months of death. In this study, we also introduced the literature-based adherence score to the Mediterranean diet (MEDI-LITE score) to compare the dietary structure (Sofi et al., 2014; Davis et al., 2015). Mediterranean diet comprises of nine food groups; each group is scored as 0, 1, 2, respectively; the total score ranges from 0 to 18. The nine groups are fruits, vegetables, beans, cereals, fish, meat and meat products, milk products, alcoholic beverages, and olive oil. The average MEDI-LITE score of 27 centenarians was 7.7 (±1.9) while the median score was eight. The obtained MEDI-LITE score of Hainan centenarians was significantly lower than that of Italians (typical Mediterranean diet area) (Barbagallo et al., 2002).

### Comparisons of Diversity and Composition of Gut Microbiota Between Hainan Centenarians and Italian Centenarians With 16S rDNA Analysis

**FIGURE 3:**
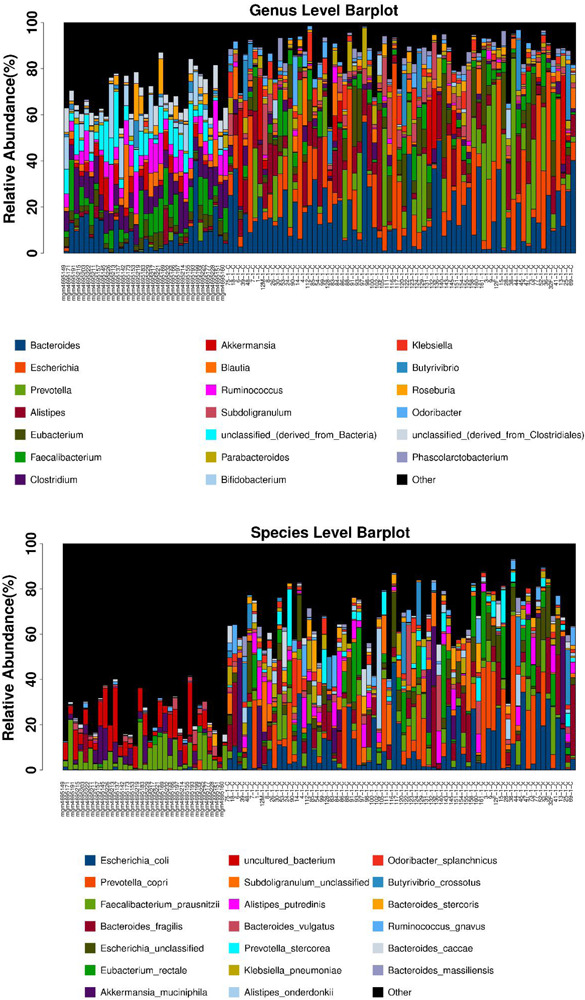
The microbial composition at the genus and species level of each samples are shown as barplot. The horizontal axis is the sample name, the vertical axis is the relative abundance ratio of genera/species. At the bottom of the barplot, the different color boxes represent different genera/species, among them the black box named other represents the sum of genera/species other than the top 20.

**FIGURE 4:**
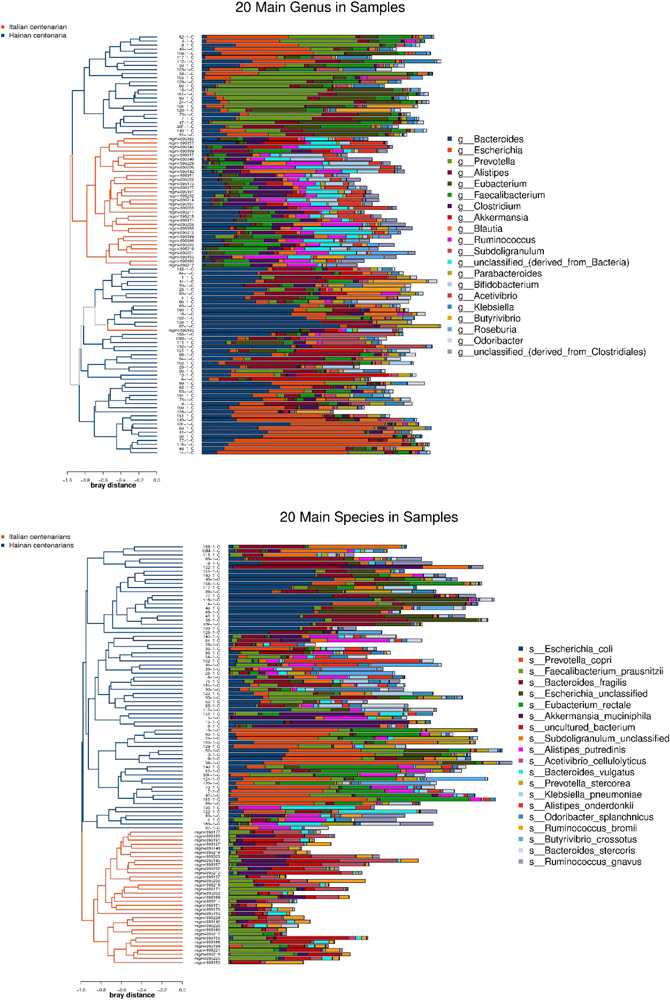
The left side of the figure shows clustering result of the samples, and the color distinguishes the groups of the samples; the middle part illustrates the relative abundance ratio of genera/species level species contained in each sample; the right side shows the genera/species level species represented by each color.

After the first round of multidisciplinary physical examination, the dominant gut microbiota at genus and species level in healthy Hainan centenarians exhibited significant differences from those of centenarians in the Mediterranean diet area of northern Italy (Figures 3, 4) (Collino et al., 2013). However, it is not clear whether the differences were associated with MEDI-LITE.

## Discussion

1. In this study, we performed multidisciplinary health status survey and performed dynamic health status tracking of centenarians in Hainan Province. The study lasted for 20 months, and 1-year mortality rate of the healthy centenarians was obtained. Centenarians represent human beings with the longest life span and constitute a valuable resource for characterization of gut microbiota in longevity population (Kong et al., 2016). In recent years, many studies on gut microbiota of centenarians have been successively published. However, most of these studies were cross-sectional studies, which did not investigate the dynamic changes in gut microbiota. Till date, no prospective cohort studies on this subject have been conducted; the most reliable methodology for a longitudinal study is to collect the fecal samples throughout the life span of centenarians. However, owing to their advanced age, healthy centenarians can experience health status changes in a relatively short time; during this period, the composition of gut microbiota may also show significant changes with the gradual decline in their health status. This finding suggests the need to monitor the changes in gut microbiota in different life stages at relatively short intervals in order to further unravel the relationship between longevity and specific gut microbiota patterns. This study is based on the nested study of CHCCS. The gut microbiota changes in the elderly toward the end of their life span were monitored during the transition from a relatively stable health state to weak state until death.

In this study, we characterized the gut microbiota changes in centenarians from health to death by performing metagenomics analysis of the gut microbiota in sequential samples obtained in the latter stages of their life. We concluded that the gut microbiota of centenarians changed sequentially in two different states; the most significant difference between each group is the predictable time inflection point which is the 7th month before death.

According to α diversity analysis, there was no significant difference between the overall gut microbiota in each group; however, the abundance of 10 bacterial species showed differences. With the 10 differential bacterial species, PCR validation analysis was performed on those centenarians who had successfully collected feces samples before and after 7 months (*n* = 17). Specific primers were not found for *Bacteroides stercoris*; therefore, PCR validation was performed on the remaining nine species. The results showed that all nine species existed in the feces of centenarians, and the absolute value of the bacteria were different; however, the overall results showed no significant difference (*P* > 0.05).

2. The age-related changes in microbiota composition have been shown to play a role in development of neurodegenerative disorders, including Parkinson’s disease (PD) and Alzheimer’s disease (AD). For example, *Akkermansia muciniphila*, which is the only known bacterial species of phylum *Verrucomicrobia* is generally recognized as a probiotic. Supplementation of this species was shown to restore the integrity of epithelial mucosa, reduce weight gain, improve glucose tolerance, and alleviate metabolic endotoxemia and inflammation in animal models of diabetes and obesity (Vaiserman et al., 2017). In our study, we observed a declining trend in the abundance of *Akkermansia muciniphila* in centenarians. These results are in keeping with the metabolic disorders that ensue with changes in physical state before death. In previous studies, the abundance of some dominant gut microbiota families (including *Bacteroides*, *Trichomonas*, and *Ruminococcaceae*) were shown to gradually decrease with increase in the age of elderly; however, there was a concomitant increase in the abundance of some other dominant bacteria families which were proposed to be related with increased longevity (Cirstea et al., 2018). The high occurrence rate and enrichment of health-related bacteria such as *Akkermansia*, *Bifidobacterium*, and *Christensenellaceae* might be associated with longevity (Biagi et al., 2016). More interestingly, we found that the abundance of a well-known probiotic, *Bifidobacterium longum* increased with the declining physical status of centenarians, which is contrary to the commonly held view. *Bifidobacterium*, one of the earliest studied probiotic, is known to play an important role in maintaining the health status of human beings. The abundance of *Bifidobacterium* is dominant in the gut microbiota of infants but is significantly decreased in the intestinal tract of adults; the decline in abundance is probably attributable to the change in dietary structure from simple to complex (Drago et al., 2012; Arboleya et al., 2016; Bonder et al., 2016). Another bacterial species, *Ruminococcus bromii*, which serve as the main degrading bacteria in human gut microbial community, exhibited an increasing trend in centenarians.
3. Comparison of data sets pertaining to centenarians with different genetic backgrounds and dietary habits suggests that gut microbiome of centenarians are related to specific genetic or living habits, including diet (Claesson et al., 2012; Wang et al., 2015; Dikongue and Segurel, 2017). This study showed significant differences in gut microbiota among centenarians in different areas. After excluding the natural bias caused by different detection and analytic methods (Zhernakova et al., 2016), it was speculated that the dietary structure and living habits also contributed to the significant differences in addition to the ethnic and genetic background (Biagi et al., 2012; Debelius et al., 2016; Requena et al., 2018). Taking centenarians in northern Italy as an example (Biagi et al., 2011, 2013), the dietary structure of that region corresponds to the typical Mediterranean diet (Saez-Almendros et al., 2013; Estruch et al., 2018). The Mediterranean diet, as a representative of healthy diet is significantly different from the western diet (Cordain et al., 2005), and is known to play an important role in the prevention of cancer, cardiovascular disease, and maintenance of longevity status. Hainan province has the highest density of longevity population in China and the raw state diet is the most common dietary structure in this province (Wang et al., 2016). Thus this dietary structure which is quite different from western diet has many similarities with the Mediterranean diet (Table 4). Interestingly, our investigation revealed that the proportion of fats in the dietary structure of Hainan centenarians (33%) is slightly higher than the standard level, which reflects the improvement in living standards and the increase in meat intake (pork as the main source of meat). However the total food intake of Hainan centenarians is not high (average intake: 1,040 kcal per day). Carbohydrates contribute about 50% of the total energy intake, which is much lower than that of Italians and Asian immigrants in north Italy (LeCroy and Stevens, 2017; Rothschild et al., 2018).

**TABLE 4:** Comparison of Mediterranean diet and Hainan diet.

**Mediterranean diet**	**Hainan diet**
1. Rich in plant foods, including fruits, vegetables, whole grains, beans, and nuts	Same
2. Simple food processing, mainly use local and seasonal fresh food	Same, mainly boiled
3. Mainly use olive oil	Other vegetable oils
4. Proportion of fat in whole diet is approximately 25–35%	Similar, <35%
5. Intake of small amounts of fish, poultry, and eggs every week	Similar
6. Eat red meat a few times per month	Mainly eat pork
7. Drink red wine	Different
8. Special food: *Portulacaoleracea* (ω-3 fatty acid)	A variety of wild vegetables

4. Although several concerted efforts were made to explore the dynamic changes of gut microbiota in centenarians, there were several challenges in this study; these included scarcity of sample resources, the dispersed residence of centenarians, and the logistical challenge of travel in Hainan (Normann et al., 2013). This 1 year cohort study needs to obtain high-quality samples in several specific periods of time. To ensure accurate analysis of data pertaining to fecal samples and to veritably reflect the gut microbiota changes in the longevity elderly, high-quality processes for follow-up, fecal sample collection, and preservation are necessary. However, all rounds of fecal samples were not successfully collected; therefore, the results may not fully correlate with the actual dynamic changes of gut microbiota in centenarians. Further study will be improved by increasing the sample size, developing standardized methods for fecal sample collection and preservation, and by increasing the duration of follow-up.
5. The novelty value of this study is that it investigated the changes in gut microbiota in dying centenarians, which has not been investigated in previous studies. Our findings and data pave the way for future studies to unravel the mystery of longevity.

## Conclusion

In this study, we investigated the gut microbiota changes of Hainan centenarians during the transition from a relatively stable health state to decline in health status and eventual death. We found that the inflection point of significant changes in gut microbiota was 7 months before death. We also found ten dominant bacterial species, which may have potential effects on physical decay of centenarians. Based on the results of previous studies, we found that compared to centenarians in northern Italy, Hainan centenarians exhibit unique characteristics of gut microbiome.

## Data Availability Statement

The datasets generated for this study are available on request to the corresponding author.

## Ethics Statement

The studies involving human participants were reviewed and approved by the Medical Ethics Committee of Hainan Branch of PLA General Hospital. The patients/participants provided their written informed consent to participate in this study.

## Author Contributions

ZL and YH analyzed the data and drafted the manuscript. CL and TW organized the data and drafted the figures. CJ, SQ, YZ, YJ, DT, CW, and SW commented the study and revised the manuscript. GS, YY, and RY designed, supervised the study, and revised the manuscript. All authors read and approved the final manuscript.

## Conflict of Interest

YH, TW, and DT were employed by the company Realbio Technology Co., Ltd.

The remaining authors declare that the research was conducted in the absence of any commercial or financial relationships that could be construed as a potential conflict of interest.
