# Supplementary material for: Metagenomics Study Reveals Changes in Gut Microbiota in Centenarians: A Cohort Study of Hainan Centenarians
Source: Front Microbiol. 2020 Jul 2;11:1474. doi: 10.3389/fmicb.2020.01474 (PMC7343713; doi:10.3389/fmicb.2020.01474)
Supplement: Supplementary file 1 [file Data_Sheet_1.PDF]

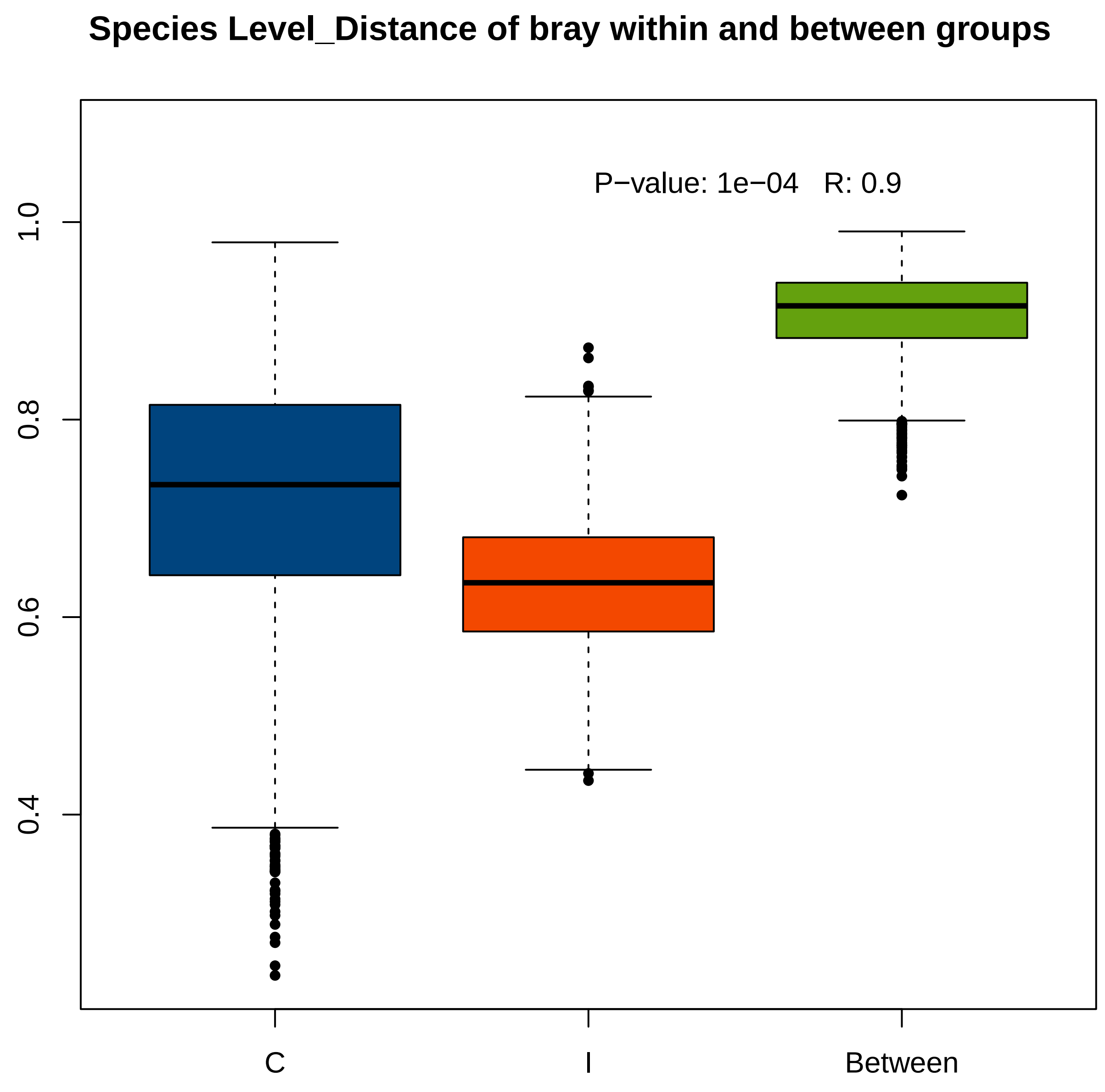

Species Level_Distance of bray within and between groups
P−value: 1e−04 R: 0.9
1.0
0.8
0.6
0.4
C
I
Between

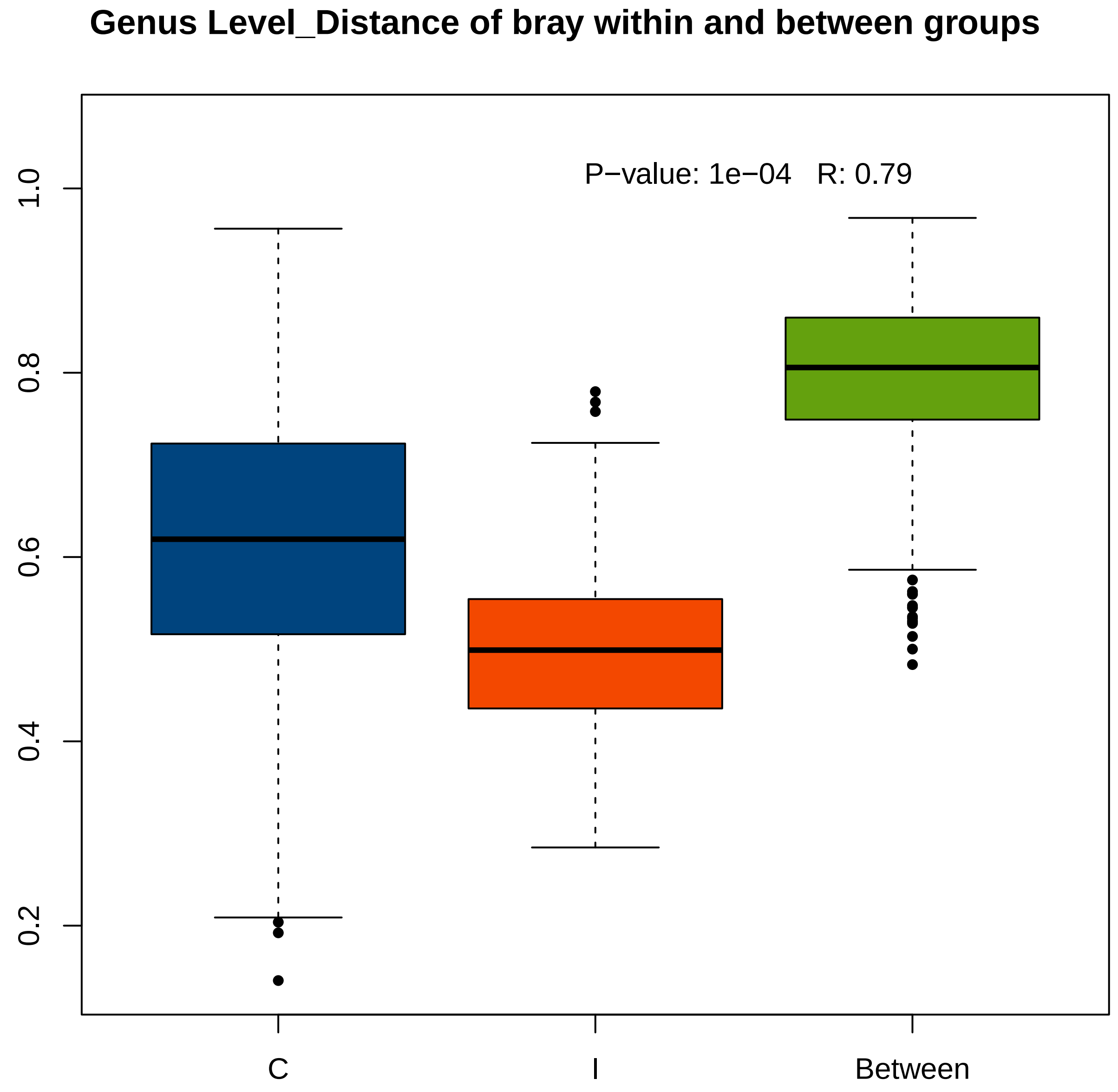
Genus Level_Distance of bray within and between groups
P−value: 1e−04   R: 0.79
1.0
0.8
0.6
0.4
0.2
C
I
Between
